# Akkermansia muciniphila improving depression-like behaviors by regulating glycerophospholipid metabolism in gut-brain axis

**DOI:** 10.3389/fphar.2026.1790866

**Published:** 2026-04-01

**Authors:** Fei He, Shunjie Bai, Jing Xie, Yongzhi Zhang, Ke Xu, Jiaolin Wang, Yi Ren, Zhe Ren, Jianjun Chen, Ying Wang, Peng Xie

**Affiliations:** 1 Institute of Neuroscience, School of Basic Medical Sciences, Chongqing Medical University, Chongqing, China; 2 Department of Laboratory Medicine, The First Affiliated Hospital of Chongqing Medical University, Chongqing, China; 3 Chongqing Key Laboratory of Emergency Medicine, Chongqing Emergency Medical Center, Chongqing University Central Hospital, Chongqing, China; 4 Department of Neurology, National Health Commission Key Laboratory of Diagnosis and Treatment on Brain Functional Diseases, The First Affiliated Hospital of Chongqing Medical University, Chongqing, China; 5 Department of Neurology, The First Affiliated Hospital of Chongqing Medical University, Chongqing, China; 6 Department of Rehabilitation, The Second Affiliated Hospital of Chongqing Medical University, Chongqing, China

**Keywords:** Akkermansia muciniphila, depression, glycerophospholipid, inflammation, lipids

## Abstract

**Background:**

Akkermansia muciniphila (AKK) is a potential probiotic. Our previous studies have shown that it could alleviate depressive-like behaviors (DLBs) in mice by inhibiting neuroinflammation in brain. To further explore its antidepressant effect, this study focused on the effects of AKK on the metabolic activities in gut-brain axis.

**Methods:**

After chronic restraint stress (CRS) depression model was successfully built, AKK was used as intervention method for 3 weeks. The gut microbiome in feces and two intestinal permeability proteins in colon (Claudin-1, Occludin) were measured, and the metabolites in feces, colon, liver, and prefrontal cortex were also measured. In addition, two inflammation-related factors in hippocampus (Free fatty acid receptors 3 (FFAR3), phosphorylated NF-κB p65 (p-p65)) were measured.

**Results:**

AKK was successfully colonized in gut of chronic restraint stress (CRS) mice. The DLBs in CRS mice receiving AKK (CRS + AKK) were significantly improved, along with the improved gut microbiome. Both Claudin-1 and Occludin in colon were significantly increased in CRS + AKK mice compared to CRS mice receiving phosphate buffer saline (PBS) (CRS + p). Metabolomics analysis indicated that AKK could significantly improve the changed lipids and lipid-like molecules in gut-brain axis of CRS mice; and function analysis using differential metabolites showed that AKK could significantly improve the disordered glycerophospholipid metabolism in feces, colon, liver, and prefrontal cortex of CRS mice. Additionally, we found that FFAR3 and phosphorylated NF-κB p65 were increased and decreased, respectively, in hippocampus of CRS + AKK mice compared to CRS + p mice.

**Conclusion:**

Our results suggested that AKK might improve the disturbances of gut microbiome, intestinal permeability, host’s lipid metabolism and inflammation levels in hippocampus. Glycerophospholipid metabolism in gut-brain axis might be the important mediator in the process of AKK producing antidepressants effects.

## 1 Introduction

Depression is a commonly disabling disease and is the leading cause of disability worldwide (Friedrich, 2017). It imposes a heavy economic burden on individuals, families, and society. Its symptoms include loss of appetite, difficulty concentrating, sleep disorders, and even suicidal thoughts (Beurel et al., 2020). Previous study has estimated that depression would become the greatest disease burden worldwide by 2030 (Zhang Y. et al., 2024). Currently, the main treatment methods for depression include medication and psychotherapy (Bennabi et al., 2015). Due to the elusive nature of its underlying causes, depression is also one of the most common and least understood diseases, resulting in the fact that the known treatment methods for depression often have poor efficacy and some side effects (Radjabzadeh et al., 2022; Cowen, 2023). Therefore, it is crucial to further explore the pathogenesis of depression.

Gut microbiome is a vast ecological community composed of bacteria, archaea, fungi, viruses and other microorganisms (Morais et al., 2020). The majority of gut microbiome in our bodies are colonized in the intestines (Cryan et al., 2019). Gut microbiome plays multiple roles, including maintaining the immune system, producing vitamins, digestion, angiogenesis, metabolite synthesis, and maintaining the integrity of the intestinal barrier (Yadav and Chauhan, 2018). Nowadays, many diseases have been confirmed to be closely related to the disturbances of gut microbiome (Vieira-Silva et al., 2019; Hou et al., 2025). At the same time, studies showed that gut microbiome had a significant impact on host’s metabolism and the functions of the central nervous system (CNS), which could affect mental health (Jiang et al., 2025; Valles-Colomer et al., 2019). For instance, a previous study showed that the imbalances in gut microbiome compositions were associated with the occurrence and development of depression (Wang G. et al., 2024; Lu et al., 2024). The specific connections between gut microbiome and CNS are known as the gut-brain axis, which involves bidirectional information exchange between gut and brain (Mayer, 2011). Previous studies reported that the bidirectional connections between the gut and the brain were based on metabolic, endocrine, neural and immune pathways (Góralczyk-Bińkowska et al., 2022; Socała et al., 2021).

Gut microbiome can produce or influence a large number of metabolites, which can enter the systemic circulation and directly or indirectly regulate the inflammatory response, neuroendocrine function and neural plasticity of CNS (Schroeder and Bäckhed, 2016). It can also affect the host’s behaviors through the gut-brain axis (Tian et al., 2022). Our previous study showed that germ-free mice receiving feces of depression patients showed obvious depression-like behaviors, along with the significant changes in blood metabolic profile (Zheng et al., 2016). These results might provide a major basis for the influence of gut microbiome on behaviors through metabolites. Evidence suggested that an increasing number of microbial metabolites have been proven to act as signaling molecules and ultimately regulate the host’s behaviors (Cheng et al., 2024).

Akkermansia muciniphila (AKK) is closely related to host’s health (Wang H. et al., 2024; Lei et al., 2023). It has been proven to be capable of restoring the balance of gut microbiome, reconstructing the integrity of intestinal mucosal barrier, and regulating the host’s immune and neuroinflammatory responses (Zhu et al., 2025). Recent studies have shown that AKK could treat certain diseases (Zhang et al., 2025; Pei et al., 2023). Over the past few years, it has been discovered that it is not only closely related to metabolic diseases, but also plays a significant role in mental illnesses, especially depression (Guo et al., 2022; Hwang et al., 2025; Guo et al., 2023). Evidence shows that gut microbiome can improve depression through their metabolites (Casertano et al., 2024; Ma et al., 2025). Our previous study found that the relative abundance of AKK was decreased in depressed subjects compared to control subjects, and AKK could significantly improve depression-like behaviors in mice via its metabolites to inhibit neuroinflammation in the hippocampus (Wang J. et al., 2025). In order to further explore the potential mechanism by which AKK exerts its antidepressant effect, this study employed a chronic restraint stress (CRS) depression model to observe the impact of AKK on the host’s metabolism. A multi-directional and multi-brain region research strategy was adopted, aiming to explore the correlation between the molecular changes in the gut and brain regions in the CRS depression model.

## 2 Materials and methods

### 2.1 CRS depression model

The mice used in this study were adult male C57 Black 6J (C57BL/6J) mice (aged 8 weeks, weighing 23 g approximately), purchased from Beijing Vital River Laboratory Animal Technology Co., Ltd. The mice were housed in a standard environment (temperature 20 °C–25 °C, humidity 40%–70%) under a 12-h light-dark cycle (lighting hours from 8:00 to 20:00). After 1 week of adaption, the mice were randomly divided into control group and CRS group; and the two groups were matched on sucrose preference (SP) and body weight (BW). According to the procedure in our previous study (Wang J. et al., 2025; Wu et al., 2020), the mice in CRS group were restrained for 6 h per day, and CON group were not disturbed; and the whole procedure lasted for 4 weeks.

### 2.2 AKK intervention and behavioral tests

After CRS depression model was successfully established, the mice in CRS group were randomly assigned into two groups: one group receiving phosphate buffer saline (PBS) (CRS + p), and one group receiving AKK (CRS + AKK). AKK was administered after 4-week of chronic stress. The dosage and method of administration (oral gavage, ATCC BAA-835, 1 × 10^9^CFU/mL, 200 μL per day) was exactly conducted according to our previous study (Wang J. et al., 2025). The entire intervention process lasted for 21 days. To prevent the influence of time differences, AKK was given at a fixed time period each day. At the end of AKK intervention, the following behavioral tests were conducted: i) sucrose preference test (SPT): each mice cage was prepared with two identical white water bottles and two identical sugar water bottles. During the adaptation period, the mice cage was placed with a white water bottle containing pure water, which lasts for 24 h. Then, it was replaced with two sugar water bottles, which lasts for 24 h. Finally, it was replaced with one sugar water bottle and one white water bottle, which lasts for 48 h, and the position of the water bottles was exchanged at the 24th hour. After a deprivation period (4 h), the mice could freely drink from the two bottles: one bottle contains pure water, and the other bottle contains a 1% sucrose solution. The consumption of the two liquids within 12 h was recorded, and the sucrose preference was calculated using the following formula: SP = [100% × Sucrose solution consumption/(Sucrose solution consumption + Pure water consumption)]. SP in SPT was used to assess the anhedonia in mice; ii) forced swimming test (FST): the experimental procedure involved placing the mice in a transparent cylindrical water tank (30 cm high) with a water depth of 20 cm (24 °C ± 1 °C). After each test, the water tank was cleaned and the water was replaced. The mice were allowed to swim for 6 min. The immobile time (IT) (analyzed automatically using the EthoVision XT 13 software) in the last 4 min was recorded, which was used to assess the level of despair in mice; and iii) open field test (OFT): after adaptation, each mouse was placed in the center of a box (44 cm × 44 cm × 44 cm). The central zone was defined as a 22 cm × 22 cm square. After each test, the box was cleaned with 75% alcohol to remove olfactory cues. The mice were allowed to explore the box for 6 min. The total distance, central distance, and central time in the last 4 min were recorded. The central time and percentage of central distance to total distance in OFT was used to assess the anxiety-like behaviors in mice.

### 2.3 16S rRNA sequencing, metabolites and intestinal permeability proteins detection

After the mice were sacrificed, the feces, colon, liver samples and prefrontal cortex of mice were collected and stored at −80 °C. The gut microbiome compositions in feces were detected using 16S rRNA sequencing technology, and the metabolites in feces, colon, liver, and prefrontal cortex were detected using liquid chromatography-mass spectrometry (LC-MS). The procedures of 16S rRNA sequencing and LC-MS were the same as we mentioned in our previous studies (Wu et al., 2020; Chen et al., 2018; Xu K. et al., 2024; Xu et al., 2025).

### 2.4 Intestinal permeability proteins and hippocampal tissue proteins detection

The operation process is as follows: First, the colon and hippocampal samples placed in a −80 °C refrigerator are taken out and separated. To prevent the samples from being contaminated or degraded, all operations are carried out rapidly under dry ice and sterile conditions. RIPA dissociation buffer, protein inhibitors, phosphatase inhibitors, EDTA, and PMSF are mixed in a fixed ratio to extract proteins. After extraction, the protein concentration is determined using the BCA kit. The extracted proteins are stored at −80 °C for refrigeration. To prevent protein degradation, the proteins should be taken out in batches for the protein blotting experiment. The protein samples are separated by electrophoresis in a 10% SDS polyacrylamide gel, and then the proteins on the gel are transferred to a polyvinylidene difluoride (PVDF) membrane. Subsequently, the PVDF membrane is blocked for 30 min with a protein-free rapid blocking buffer (Epizyme, PS108P). After blocking is completed, the membrane is cut according to the molecular weight of the target protein and the internal control. Then, the bands and the primary antibody are incubated overnight (16–24 h) at 4 °C. The primary antibody for detecting intestinal permeability proteins is GAPDH (60004-1-Ig, Proteintech), Occludin (1:1000, WL01996, Wanlai Biology), and Claudin-1 (1:1000, WL03073, Wanlai Biology); the primary antibody for detecting hippocampal tissue proteins is β-actin (66009-1-Ig,Proteintech), Free fatty acid receptors 3 (FFAR3) (1:1,000, ab236654,abcam), and phosphorylated NF-κB p65 (p-p65) (1:1,000, WL02169, Wanlai Biology). The next day, the membrane is incubated with the secondary antibody (1:10,000) for 1.5 h. The protein bands are stained using the enhanced chemiluminescence protein blotting kit according to the kit instructions. The gray values of the protein bands are analyzed using ImageJ software.

### 2.5 Statistical analysis

The data analysis was analyzed using R Studio (version 4.0.5) and online platform of Majorbio Cloud Platform (www.majorbio.com). Student’s t-test, one-way analysis of variance (ANOVA), Spearman correlation analysis and non-parametric tests were used when appropriate. In one-way ANOVA, if a significant difference was found, a least significant difference (LSD) or Tamhan’s T2 post-hoc analysis was performed to explore which two groups differed significantly according to the equal variance criterion. Principal co-ordinates analysis (PCoA) was used to explore the differential species that were affected by AKK, and orthogonal partial least squares discriminant analysis (OPLS-DA) was used to explore the differential metabolites that were affected by AKK. The validity of OPLS-DA model was evaluated through permutation testing (n = 300) and by examining the Q^2^ and R^2^ values. Function analysis using differential species or metabolites was conducted based on Kyoto Encyclopedia of Genes and Genomes (KEGG) database. Here, we selected Benjamini and Hochberg False Discovery method to do multiple testing corrections. The adjusted p < 0.05 was considered statistically significant.

## 3 Results

### 3.1 Behavior characteristics in different groups

**FIGURE 1:**
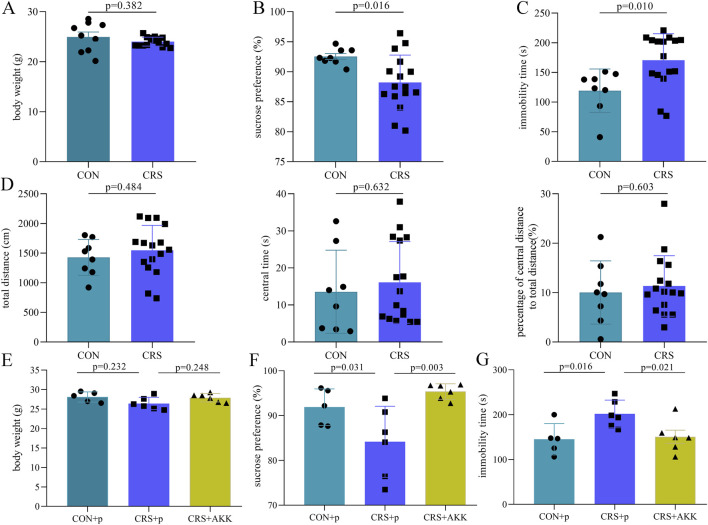
AKK improved the depressive-like behaviors in mice: **(A)** body weight was similar between the two groups after CRS procedure; **(B)** CRS mice had significantly lower sucrose preference compared to CON mice; **(C)** CRS mice had significantly higher immobility time compared to CON mice; **(D)** total distance, central time and percentage of central distance to total distance were similar between the two groups; **(E)** body weight was similar among the three groups after AKK intervention; **(F)** sucrose preference was significantly improved after AKK intervention; **(G)** immobility time was significantly improved after AKK intervention. CON, control; CRS, chronic resistant stress; p, phosphate buffer saline; AKK, Akkermansia muciniphila.

After CRS procedure, BW was not significantly different (F = 19.149, t = 0.920, p = 0.382, effect size = 0.757) between CON mice (n = 9) and CRS mice (n = 16) (Figure 1A). But, compared to CON mice (n = 8, one mice dead during SPT), CRS mice (n = 16) had significantly lower SP (F = 6.576, t = 2.606, p = 0.016, effect size = 0.668; Figure 1B) and higher IT (F = 1.220, t = −2.812, p = 0.010, effect size = 1.029; Figure 1C), suggesting that there were obvious depression-like behaviors in CRS mice. In OFT, the total distance (F = 0.676, t = −0.713, p = 0.484, effect size = 1.001), central time (F = 0.014, t = −0.485, p = 0.632, effect size = 1.023) and percentage of central distance to total distance (F = 0.110, t = −0.527, p = 0.603, effect size = 1.002) were similar between the two groups, suggesting that there was no anxiety-like behavior in CRS mice (Figure 1D).

After AKK intervention, BW was not significantly different among the three groups (randomly, 5 CON + p mice, 6 CRS + p mice and 6 CRS + AKK mice; Figure 1E). Meanwhile, compared to CRS + p mice, both CON + p mice (F = 6.946, p = 0.031, effect size = 1.061) and CRS + AKK mice (F = 6.949, p = 0.003, effect size = 2.943) had significantly higher SP; and SP was similar between CON + p mice and CRS + AKK mice (Figure 1F). Meanwhile, after AKK intervention, compared to CRS + p mice, both CON + p mice (F = 4.852, p = 0.016, effect size = 0.783) and CRS + AKK mice (F = 4.852, p = 0.021, effect size = 0.951) had significantly lower IT; and IT was similar between CON + p mice and CRS + AKK mice (Figure 1G). These results demonstrated that AKK could significantly improve the depression-like behaviors in mice.

### 3.2 Effects of AKK on gut microbiome compositions

**FIGURE 2:**
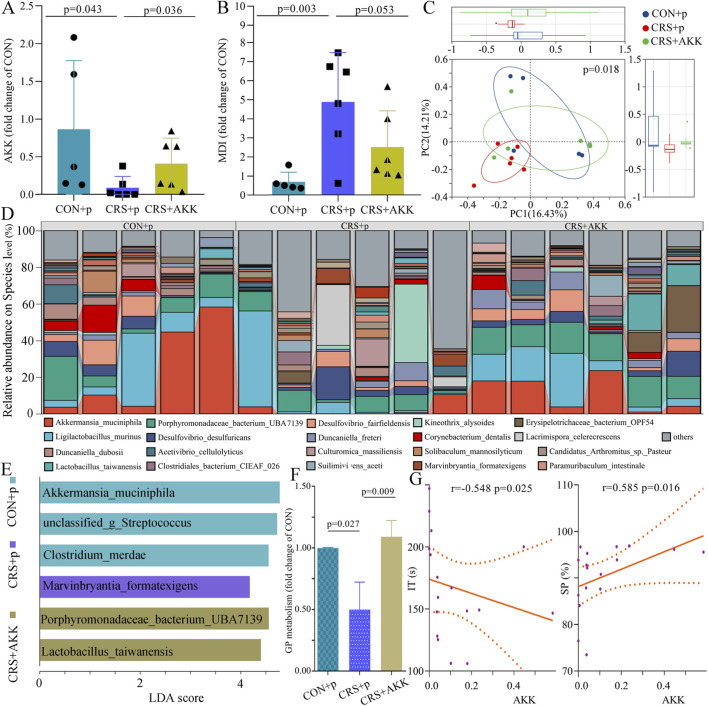
Gut microbiome compositions among the three groups: **(A)** AKK was successfully colonized in CRS mice; **(B)** microbial dysbiosis index in three groups; **(C)** the results of PCoA indicated the significant differences on gut microbiome compositions among the three groups; **(D)** the relative abundances on species level in three groups; **(E)** the six differential species identified using linear discriminant analysis (LDA) effect size (LEfSe); **(F)** AKK could improve the disordered GP metabolism in CRS mice; **(G)** AKK was closely related with depressive-like behaviors. CON, control; CRS, chronic resistant stress; p, phosphate buffer saline; AKK, Akkermansia muciniphila; SP, sucrose preference; IT, immobility time; MDI, microbial dysbiosis index; GP, glycerophospholipid metabolism.

As shown in Figure 2A, compared to CRS + p mice, CON + p mice (p = 0.043) had significantly higher levels of AKK, suggesting that AKK was significantly decreased in CRS mice; compared to CRS + p mice, CRS + AKK mice (p = 0.036) had significantly higher levels of AKK, suggesting that AKK was successfully colonized in CRS mice. The microbial dysbiosis index (MDI) showed that gut microbiome was significantly disordered in CRS + p mice (p = 0.003), and AKK could improve this situation (Figure 2B). The values of Shannon (p = 0.214), Simpson (p = 0.320), Chao1 (p = 0.407) were similar among the three groups, suggesting the similar alpha diversity between the different groups. The PCoA model on species showed that gut microbiome compositions among the three groups were significantly different (p = 0.018, Figure 2C). The relative abundances on species level were shown in Figure 2D. Using linear discriminant analysis (LDA) effect size (LEfSe), six species with statistically significant and biologically consistent differences among the three groups were identified (LDA >2.0, p < 0.05, Figure 2E; Supplementary Table S1 in Supplementary Material S1). The function prediction using these six species showed that glycerophospholipid metabolism was significantly disordered in CRS + p mice (vs. CON + p mice, p = 0.027), and this situation could be improved by AKK (CRS + p mice vs. CRS + AKK mice, p = 0.009; CON + p mice vs. CRS + AKK mice, p = 0.664; Figure 2F). Spearman correlation analysis showed that AKK was significantly related with IT (r = −0.548, p = 0.025) and SP (r = 0.585, p = 0.016; Figure 2G). These results indicated that glycerophospholipid metabolism might be the mediator of AKK improving depression-like behaviors.

### 3.3 Effects of AKK on intestinal permeability proteins

**FIGURE 3:**
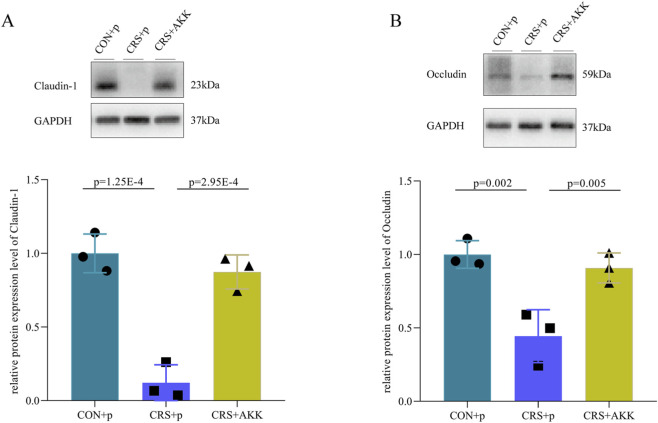
AKK improves the intestinal permeability of mice with CRS: **(A)** the level of Claudin-1 was significantly decreased in CRS + p mice, but significantly improved in CRS + AKK mice; **(B)** the level of Occludin was significantly decreased in CRS + p mice, but significantly improved in CRS + AKK mice. CON, control; CRS, chronic resistant stress; p, phosphate buffer saline; AKK, Akkermansia muciniphila.

The levels of Claudin-1 and Occludin in colon were measured here. We found that the levels of Claudin-1 in CRS + p mice were significantly lower compared to CON + p mice (p = 1.25E-4, Figure 3A), suggesting the impaired intestinal permeability; and the levels of Claudin-1 were significantly increased in CRS + AKK mice compared to CRS + p mice (p = 2.95E-4, Figure 3A). Meanwhile, we found that the levels of Occludin in CRS + p mice were significantly lower compared to CON + p mice (p = 0.002, Figure 3B), also suggesting the impaired intestinal permeability; and the levels of Occludin were significantly increased in CRS + AKK mice compared to CRS + p mice (p = 0.005, Figure 3B). The levels of these two proteins were similar between CON + p mice and CRS + AKK mice. These results suggested that AKK might be capable of improving the impaired intestinal permeability.

### 3.4 Effects of AKK on fecal metabolites

**FIGURE 4:**
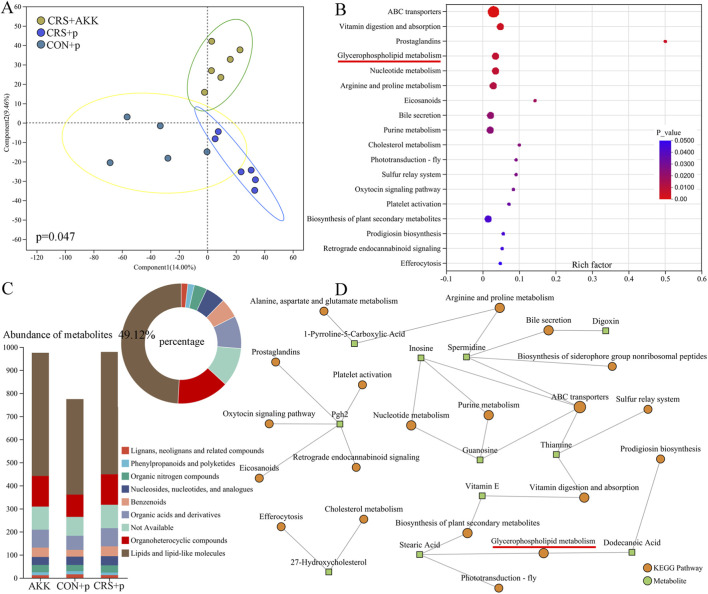
AKK affected fecal metabolites in CRS mice: **(A)** OPLS-DA model built with fecal metabolites among the three groups; **(B)** 18 differential pathways was identified using the differential metabolites; **(C)** the classification of differential metabolites; **(D)** the interaction of differential pathways and differential metabolites. CON, control; CRS, chronic resistant stress; p, phosphate buffer saline; AKK, Akkermansia muciniphila; KEGG, Kyoto Encyclopedia of Genes and Genomes; OPLS-DA, orthogonal partial least squares discriminant analysis.

As shown in Figure 4A, the results of OPLS-DA showed that there were divergent fecal metabolic phenotypes among the three groups (p = 0.047, Figure 4A). Both permutation testing (Supplementary Figure S1A in Supplementary Material S1) and values of Q^2^ (0.663) and R^2^ (0.991) showed that the built OPLS-DA model was valid and not overfitting. Using variable importance in projection (VIP >1.0) and p < 0.05, 76 differential metabolites were identified (Supplementary Table S2 in Supplementary Material S1). Function analysis using these differential metabolites identified 18 differential pathways (Supplementary Table S2), including glycerophospholipid metabolism (p = 0.007, Figure 4B). There were 10 glycerophospholipids-related differential metabolites. The annular chart showed that these differential metabolites mainly belonged to Lipids and lipid-like molecules, and the stacked column of metabolite abundances showed that the abundances of these differential metabolites were higher than the abundances of metabolites belonged to other class (Figure 4C). The interaction of differential pathways and differential metabolites were displayed in Figure 4D. These results indicated that AKK might significantly affect the metabolic phenotype in CRS mice, especially lipids and lipid-like molecules.

### 3.5 Effects of AKK on colon and liver metabolites

**FIGURE 5:**
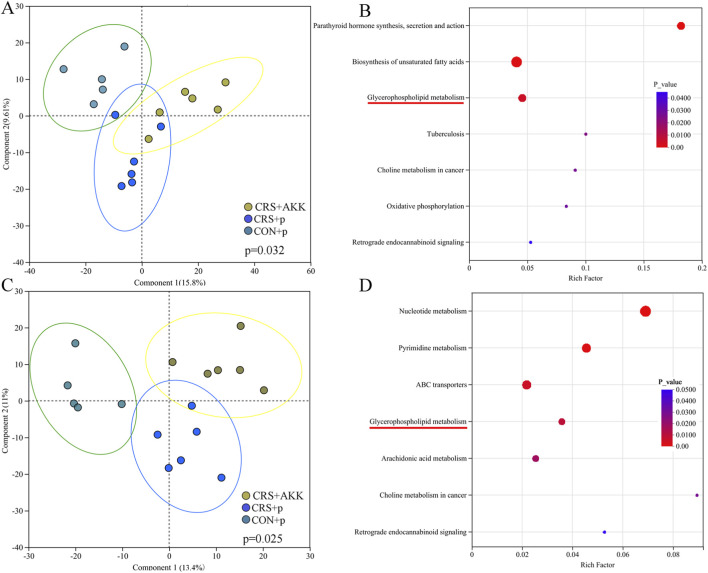
AKK affected metabolites in colon and liver of CRS mice: **(A)** OPLS-DA model built with metabolites in colon among the three groups; **(B)** seven differential pathways was identified using the differential metabolites; **(C)** OPLS-DA model built with metabolites in liver among the three groups; **(D)** seven differential pathways was identified using the differential metabolites. CON, control; CRS, chronic resistant stress; p, phosphate buffer saline; AKK, Akkermansia muciniphila; OPLS-DA, orthogonal partial least squares discriminant analysis.

Firstly, the metabolites in colon were analyzed. The results of OPLS-DA showed that there were divergent metabolic phenotypes among the three groups (p = 0.032, Figure 5A). Both permutation testing (Supplementary Figure S1B in Supplementary Material S1) and values of Q^2^ (0.647) and R^2^ (0.724) showed that the built OPLS-DA model was valid and not overfitting. Using VIP >1.0 and p < 0.05, 51 differential metabolites were identified (Supplementary Table S3 in Supplementary Material S1). Function analysis using these differential metabolites identified seven differential pathways (Supplementary Table S3), including glycerophospholipid metabolism (p = 0.005, Figure 5B). There were six glycerophospholipids-related differential metabolites. Secondly, the metabolites in liver were analyzed. The results of OPLS-DA showed that there were divergent metabolic phenotypes among the three groups (p = 0.025, Figure 5C). Both permutation testing (Supplementary Figure S1C in Supplementary Material S1) and values of Q^2^ (0.616) and R^2^ (0.990) showed that the built OPLS-DA model was valid and not overfitting. Using VIP >1.0 and p < 0.05, 65 differential metabolites were identified (Supplementary Table S4 in Supplementary Material S1). Function analysis using these differential metabolites identified seven differential pathways (Supplementary Table S4), including glycerophospholipid metabolism (p = 0.009, Figure 5D). There were 11 glycerophospholipids-related differential metabolites. The differential metabolites in both colon and liver also mainly belonged to lipids and lipid-like molecules.

### 3.6 Effects of AKK on prefrontal cortex metabolite

**FIGURE 6:**
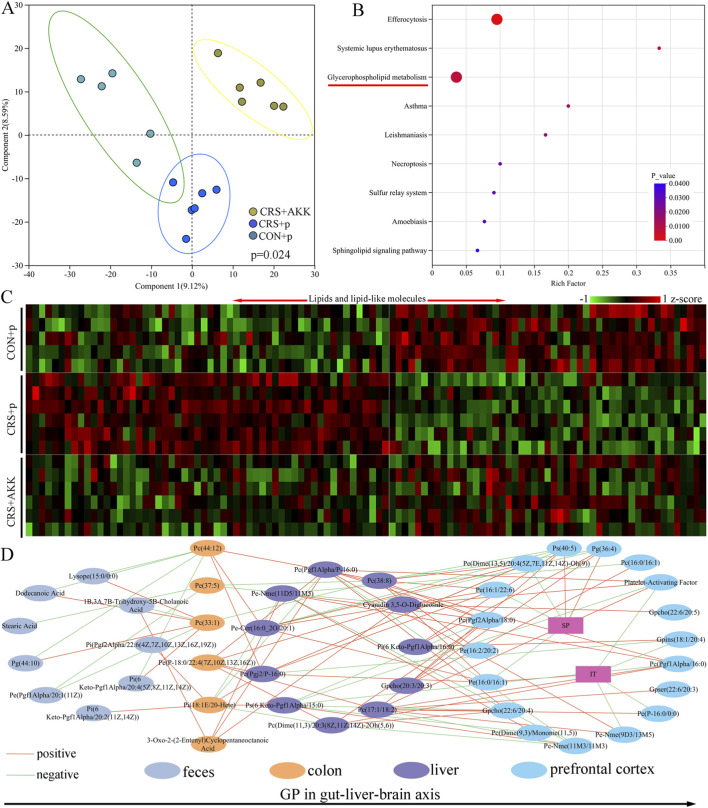
AKK affected metabolites in gut-liver-brain of CRS mice: **(A)** OPLS-DA model built with metabolites in prefrontal cortex among the three groups; **(B)** nine differential pathways was identified using the differential metabolites; **(C)** heatmap of differential metabolites in the gut-liver-brain axis; **(D)** close relationships of glycerophospholipids-related differential metabolites in gut-liver-brain axis; CON, control; CRS, chronic resistant stress; p, phosphate buffer saline; AKK, Akkermansia muciniphila; OPLS-DA, orthogonal partial least squares discriminant analysis.

The metabolites in prefrontal cortex were analyzed. The results of OPLS-DA showed that there were divergent metabolic phenotypes among the three groups (p = 0.024, Figure 6A). Both permutation testing (Supplementary Figure S1D in Supplementary Material S1) and values of Q^2^ (0.634) and R^2^ (0.931) showed that the built OPLS-DA model was valid and not overfitting. Using VIP >1.0 and p < 0.05, 61 differential metabolites were identified (Supplementary Table S5 in Supplementary Material S1), and these differential metabolites mainly belonged to Lipids and lipid-like molecules. Function analysis using these differential metabolites identified nine differential pathways (Supplementary Table S5), including glycerophospholipid metabolism (p = 0.007, Figure 6B). There were 18 glycerophospholipids-related differential metabolites. The heat-map built using the differential metabolites in the gut-liver-brain axis showed a consistent clustering pattern within the individual groups, suggesting that AKK could significantly improve the disordered metabolic levels in CRS mice (Figure 6C). Spearman correlation analysis showed that there were close relationships between peripheral and central glycerophospholipids-related differential metabolites (Figure 6D). Based on the results of feces, colon, liver and prefrontal cortex, we found that AKK could consistently affected glycerophospholipid metabolism in gut-brain axis.

### 3.7 Effects of AKK on inflammatory-related molecules in the hippocampus

**FIGURE 7:**
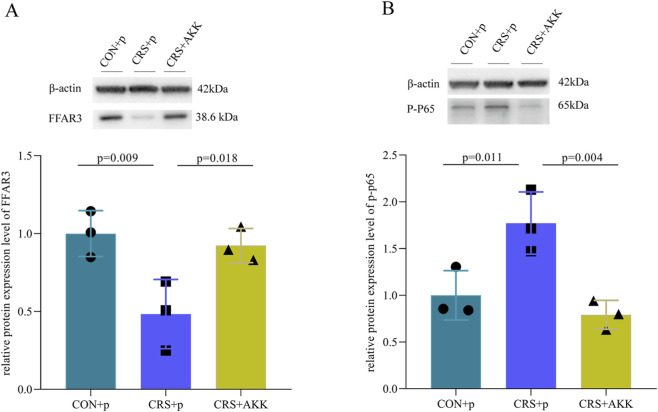
AKK can improve the expression of inflammation-related molecules in the hippocampus region of CRS mice: **(A)** In CRS + p mice, the levels of FFAR3 were significantly decreased compared to CON + p mice, while in CRS + AKK mice, they were significantly improved; **(B)** In CRS + p mice, the level of p-p65 was significantly elevated compared to CON + p mice, while in CRS + AKK mice, it was significantly improved. CON, control group; CRS, chronic resistant stress; p, phosphate-buffered saline; AKK, Akkermansia muciniphila.

The levels of FFAR3 and p-p65 in hippocampus were measured here. We found that the levels of FFAR3 in CRS + p mice were significantly lower compared to CON + p mice (p = 0.009, Figure 7A), and the levels of FFAR3 were significantly higher in CRS + AKK mice compared to CRS + p mice (p = 0.018, Figure 7A). Meanwhile, we found that the levels of p-p65 in CRS + p mice were significantly higher compared to CON + p mice (p = 0.011, Figure 7B), and the levels of p-p65 were significantly lower in CRS + AKK mice compared to CRS + p mice (p = 0.004, Figure 7B). The levels of these two proteins were similar between CON + p mice and CRS + AKK mice. These results suggested that AKK might be able to improve the inflammation levels in the hippocampus of CRS mice.

## 4 Discussion

In this study, we found that AKK was decreased in mice with depression-like behaviors and AKK could improve depression-like behaviors in mice, which was consistent with our previous findings (Wang J. et al., 2025). Meanwhile, we also found that AKK could improve the intestinal permeability of CRS mice and significantly affect the metabolic phenotypes in peripheral and central tissues. Further analysis showed that the differential metabolites mainly belonged to lipids and lipid-like molecules, and the significantly affected glycerophospholipid metabolism was simultaneously found in feces, colon, liver and prefrontal cortex. These results indicated that glycerophospholipid metabolism was an important mediator in the process of AKK producing antidepressant effects.

Current research indicated that AKK could improve depressive phenotypes via metabolites (Khalili et al., 2025; Sun et al., 2023). Cheng et al. reported that AKK could significantly reduce anxiety and depression-like behaviors in mice via regulating 5-hydroxytryptamine (Cheng et al., 2022). Our previous study found that AKK could improve depression-like behaviors in mice via regulating short-chain fatty acids (SCFAs) in feces (Wang J. et al., 2025). In addition, previous studies reported that AKK could improve ulcerative colitis and chemotherapy-induced cardiotoxicity through mediating host intestinal permeability proteins (Claudin-1 and Occludin) (Zhang H. et al., 2024; Lin et al., 2025). Interestingly, in this study, we found that Occludin and Claudin-1 were significantly decreased in CRS mice, while AKK could significantly increase the levels of these two intestinal permeability proteins. The significantly decreased level of Claudin-1 in colon of mice with depression-like behaviors was also observed in our previous study (Wu et al., 2025). These results indicated that the antidepressant effects of AKK might be closely related to its effects on improving intestinal permeability.

It is worth noting that depression is not confined to a single brain region (Nie et al., 2024). We also tested molecules related to inflammation in the hippocampus. The results showed that the expression of FFAR3 was down-regulated, while the expression of p-p65 was up-regulated in CRS + P model mice. After AKK intervention, the expression of these two molecules were significantly improved in CRS mice. Interestingly, FFAR3 is a key receptor for short-chain fatty acids (SCFAs) (Wang Y. et al., 2025). In a depressed state, the expression of FFAR3 decreases, thereby weakening the inhibitory effect on NF-κB pathway and leading to an increase in the level of the core marker p-p65 of the NF-κB pathway, thereby triggering neuroinflammation in brain regions (Ma et al., 2025; Wang Q. et al., 2025). Additionally, our previous study found that AKK could significantly increase FFAR2 and non-significantly increase FFAR3, and FFAR2 antagonist could counteract the antidepressant effects of AKK (Wang J. et al., 2025). Therefore, the above results indicated that AKK might exert an antidepressant effects via SCFAs receptors to improve neuroinflammation in hippocampus.

Mechanistically, gut dysbiosis has some negative effects on intestinal barrier integrity, allowing lipopolysaccharide and bacteria to enter circulation (Zhang Y. D. et al., 2024; Chen et al., 2024; Jia et al., 2025). This phenomenon will trigger systemic inflammation, such as the increased levels of interleukin-6 and tumor necrosis factor-alpha, which can directly undermine blood-brain barrier (BBB) tight junctions (Prajapati et al., 2025; Munshi et al., 2025). In turn, the compromised BBB enables the infiltration of inflammatory mediators into the brain, where they activate microglia and trigger neuroinflammation, thereby disrupting neuronal function and synaptic plasticity. In parallel, the gut-brain neuronal axis operates via the vagus nerve (Wang Z. et al., 2025). Enteroendocrine and enterochromaffin cells can detect microbial metabolites and rapidly transmit these signals to brainstem nuclei, which project to emotion-regulating centers, providing a direct neural pathway for gut microbes to influence mood and behavior. These findings provide a solid foundation for microbiota-based interventions in the treatment of depression.

Many studies reported that gut microbiome was involved in the onset of depression-like behaviors via medicating host’s metabolism (Zheng et al., 2016; Nie et al., 2024). Glycerophospholipid, as the core component of cell membrane and a precursor of signaling molecules, plays a crucial role in the functions of CNS. Bisle et al. found that glycerophospholipid might be potential targets for further research and intervention in depression (Bisle et al., 2025). Cao et al. reported that gut microbiome might cause depression-like behaviors via modulating central glycerophospholipid metabolic homeostasis (Cao et al., 2025). Using a depression model, we found that glycerophospholipid metabolism was closely related to the onset of depression-like behaviors in mice (Tian et al., 2022; Xie et al., 2023). Here, we found that glycerophospholipid metabolism in both peripheral and central tissues was significantly affected by AKK. Meanwhile, a previous study reported that AKK could regulate lipid metabolism in liver (Hu et al., 2025). Considering these findings, we suggested that AKK might produce antidepressant effects via affecting host’s glycerophospholipid metabolism.

Evidence shows that there were close relationships between glycerophospholipid metabolism and depression. According to previous findings (Yan et al., 2025; Wu et al., 2024; Li et al., 2025; Jansen et al., 2024), changes in glycerophospholipids may regulate depression-like behaviors through multiple mechanisms: firstly, membrane phospholipids, such as phosphatidylcholine (PC) and phosphatidylethanolamine (PE), can affect membrane fluidity, thereby regulating the functions of receptors and affecting signal transduction. Secondly, phosphatidylserine can participate in synaptic vesicle circulation, and its dysregulation will impair the release of neurotransmitters. Thirdly, the disturbances of glycerophospholipids metabolism can induce neuroinflammation. Fourthly, specific glycerophospholipids, such as PC and PE can serve as signaling lipid mediators, and their levels are significantly correlated with depression-like behaviors. Here, we found that glycerophospholipid metabolism might be the important mediator in the process of AKK regulating intestinal permeability and inflammation levels in CRS mice.

Additionally, CRS depression model was selected here for several reasons: i) it is a validated model of psychological stress; ii) it is extensively validated for its ability to induce core behavioral and physiological phenotypes of depression (Lebedeva et al., 2020); iii) it only includes simple physical restraint, allowing for a clearer and simpler interpretation of the results; and iv) the procedure of this model is simple, highly reproducible across laboratories. However, other commonly used stress models which might show more clinical similarities to depression, such as single prolonged stress and novel stress re-stress mode (Lebedeva et al., 2017), should be considered. These models can serve as effective alternatives for constructing depression models.

In this study, we observed a significant reduction in AKK abundance in CRS mice, aligning with previous findings that chronic stress could alter gut microbiota composition (Lei et al., 2023). The decrease in AKK under CRS conditions may be attributed to stress-induced disruption of the intestinal mucus layer, as AKK relies on mucin as its primary energy source (Lei et al., 2023; Xu Q. et al., 2024). Chronic stress can activate hypothalamic-pituitary-adrenal axis and then increase glucocorticoid levels, which can directly impact gut barrier integrity and reduce mucus production, thereby producing negative effects on AKK (Misera et al., 2024). Our findings both aligned with and extended the existing microbiome studies. Consistent with a recent meta-analysis demonstrating that AKK supplementation could alleviate depression-like behaviors and reduce neuroinflammation in rodent models (Khalili et al., 2025), our results supported the important role of AKK against stress-induced depression. Meanwhile, our study indicated that endogenous AKK reduction during chronic stress might serve as a key factor contributing to depression-like behaviors. This bidirectional perspective could provide a more comprehensive understanding of microbiota-gut-brain axis in depression pathophysiology and highlight the potential of targeting AKK for both prevention and treatment strategies.

Several limitations need to be mentioned here. Firstly, this study was conducted only on mice. Future research should combine samples from clinical patients with depression to conduct a correlation study between animal experiments and clinical samples. Secondly, this study only investigated two brain regions, the hippocampus and the prefrontal lobe. Future research should collect more brain regions to further explore the potential mechanism by which AKK improves depressive-like behaviors. Thirdly, we only discussed the antidepressant effect of one AKK dose in this study. Future research should further evaluate the antidepressant effects of other AKK doses. Fourthly, possibly because 3 weeks might be sufficient for self-remission of symptoms in rodent models (Seewoo et al., 2020; Prajapati et al., 2020), we finally succeeded in establishing a stable model after three attempts in this study. Therefore, future studies should further optimize experimental parameters-such as increasing the AKK dose, administering it twice daily-to shorten the intervention period for improving the success rate of model construction. Fifthly, we did not consider BW when analyzing IT in FST. Considering that buoyancy was directly related to density, it might be appropriate to analyze the effects of BW on IT, such as IT(s)/BW(g). Sixthly, we only relied on protein expression levels to assess intestinal permeability. Future studies should incorporate functional validation, such as the fluorescein isothiocyanate (FITC)-dextran assay. Seventhly, we conducted metabolomics in the prefrontal cortex and inflammatory detection in the hippocampus here. It might be more appropriate to conduct both analyses in the same brain region. Eighthly, SCFAs were not measured in the current study, and relied solely on previous findings. Direct measurement of SCFAs would significantly enhance the mechanistic interpretation. Finally, we found that AKK could significantly increase FFAR3 here (using Western blot), but non-significantly increase in our previous study (using enzyme-linked immunosorbent assay) (Wang J. et al., 2025). These inconsistent results might have resulted from differences in sample sizes or detection methods.

## 5 Conclusion

In conclusion, our research found that gut microbiome and two intestinal permeability proteins in the CRS mice were disordered, and the levels of inflammatory-related factors in the hippocampus were also affected. After receiving AKK intervention, the depression-like behaviors of CRS mice were significantly improved, as well as the disordered gut microbiome and intestinal permeability proteins; and the changed levels of FFAR3 and p-p65 in hippocampus were also improved. In addition, we also found that AKK could significantly improve the metabolic phenotype of CRS mice, especially glycerophospholipid metabolism. Our results could be helpful for further exploring the mechanisms of AKK producing antidepressant effects.

## Data Availability

The datasets generated during the current study were available in online repositories. The names of the repository/repositories and accession number(s) can be found below: http://www.ncbi.nlm.nih.gov/bioproject/1400281.
